# Comparative Study of Popular Objective Functions for Damping Power System Oscillations in Multimachine System

**DOI:** 10.1155/2014/549094

**Published:** 2014-04-06

**Authors:** Naz Niamul Islam, M. A. Hannan, Hussain Shareef, Azah Mohamed, M. A. Salam

**Affiliations:** ^1^Department of Electrical, Electronic and Systems Engineering, Faculty of Engineering and Built Environment, Universiti Kebangsaan Malaysia, 43600 Bnagi, Selangor, Malaysia; ^2^Electrical and Electronic Engineering Program, Faculty of Engineering, Institut Teknologi Brunei (Engineering and Technology University), Jalan Tungku Link, Gadong BE1410, Brunei Darussalam

## Abstract

Power oscillation damping controller is designed in linearized model with heuristic optimization techniques. Selection of the objective function is very crucial for damping controller design by optimization algorithms. In this research, comparative analysis has been carried out to evaluate the effectiveness of popular objective functions used in power system oscillation damping. Two-stage lead-lag damping controller by means of power system stabilizers is optimized using differential search algorithm for different objective functions. Linearized model simulations are performed to compare the dominant mode's performance and then the nonlinear model is continued to evaluate the damping performance over power system oscillations. All the simulations are conducted in two-area four-machine power system to bring a detailed analysis. Investigated results proved that multiobjective D-shaped function is an effective objective function in terms of moving unstable and lightly damped electromechanical modes into stable region. Thus, D-shape function ultimately improves overall system damping and concurrently enhances power system reliability.

## 1. Introduction

Reliability and security of multimachine power system are achieved through numerous controllers. Design of robust damping controllers for the interconnected power system is very sensitive in order to suppress oscillations [1]. Power system oscillation is very harmful to the overall interconnected system. Therefore, proper and adequate damping is required to prevent damages caused by oscillations [1]. Damping power system oscillations are performed by means of power system stabilizers (PSSs) installed in the generator's excitation system and flexible AC transmission system (FACTS) devices installed in transmission line for fast acting damping performance [2–4]. The damping performance by PSS and FACTS depends on proper selection of its controller parameters [3]. The design of PSS and FACTS controllers usually is achieved in the linearized model by minimizing or maximizing an objective function using metaheuristic optimization algorithms [5–9]. Improving damping performance has been proposed by many superior metaheuristic algorithms using inconsistent objective functions [5, 9–13]. However, metaheuristic algorithms are used to minimize or maximize an objective function that correspond optimized parameters under several system operating conditions [11, 14]. Therefore, selection of objective function may influence the overall controller optimization problem and that consequently affects existing system reliability.

In the last few years, many approaches to formulate the objective functions have been used in the damping controller design problem. That may be categorized into two types: (i) single objective functions and (ii) multiobjective functions. Application of single objective functions is presented either in terms of damping factors or damping ratios of electromechanical modes. In many literatures, the worst damping factor (largest value) has been selected and then the aim of optimization was set to minimize it [5–8]. On the other hand, damping ratio is also important to limit the overshoot of oscillations which ultimately improves damping performance of controllers. Therefore, the lowest damping ratio has been determined and controller parameters were optimized in such a way to maximize it in [9]. However, system damping depends on the performance of dominants electromechanical modes rather than a single mode only. So, a single objective function known as comprehensive damping index (CDI) has been proposed in [10] that includes dominant modes. As both of the damping factor and damping ratio contribute to enhance damping performance, significance of multiobjective functions is realized in numerous researches [11–19]. In multiobjective techniques, the formulation to place electromechanical modes into a D-shaped region is very popular. However, different approaches have been applied to form D-shaped stability area. In [11, 14–18], a technique to form D-shaped stability zone is discussed based on the expected damping factor and ratio for dominant modes. In that objective function, only selected dominant modes are considered into optimization that do not have expected damping factor or damping ratio. Another approach to set up D-shaped region has been claimed in [12, 19] by the algebraic sum of the worst damping factor and ratio. Moreover, a different arrangement is reported in [13] in order to form D-shaped sector.

Application of the objective function with different formulations has been proposed for the design of robust damping controllers. As an objective function formulation is quite sensitive to damping controller parameter optimization, it is very significant to evaluate their comparative performance under a common base. Therefore, this research comes forward to conduct a comparative and comprehensive investigation to evaluate the existing objective functions for damping controller design in multimachine power system. Popular objective functions are considered to use with a metaheuristic optimization algorithm under a base setting. Later on, the analysis is extended to assess performances in linearized model and nonlinear model of the power system.

## 2. Power System and Damping Controller

**Figure 1:**
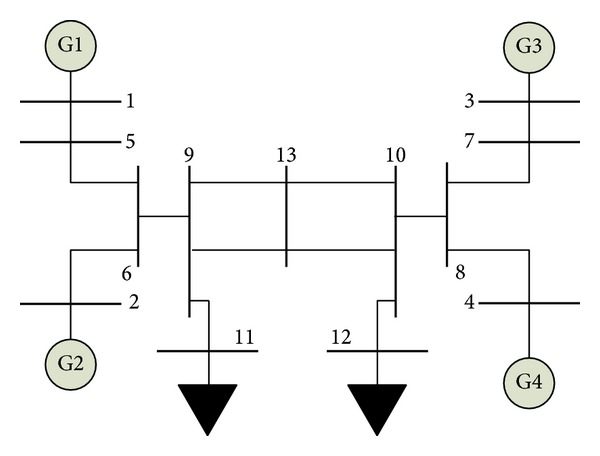
Single line diagram of two-area four-machine power system.

Two-area four-machine power system shown in Figure 1 is a benchmark system to study power system oscillations [1, 2]. The performance of local and interarea modes of oscillations can be observed easily in this system [2]. Therefore, it is adopted to design damping controllers by means of PSSs. The overall system has 13 buses, two load areas, and four synchronous generators equipped with similar components. The excitation system is the IEEE type ST1 exciter. The detail system data are taken from [2].

**Figure 2:**
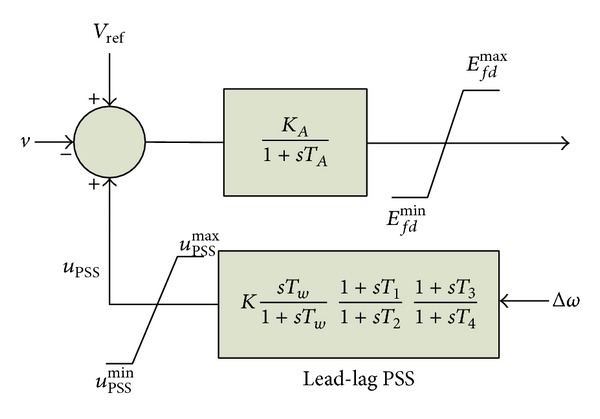
IEEE type ST1 excitation system with power system stabilizer.

A PSS is added with each synchronous generator to provide an additional damping torque if system tempts to become unstable for oscillations. All PSSs are conventional PSS (CPSS) which consists of two-stage lead-lag compensation blocks with one gain and one washout block. The rotor speed deviation (Δ*ω*) is the input of PSS. IEEE type ST1 excitation system with PSS is presented in Figure 2, where *T*_*w*_ is the washout time constant and it is set to 10 s for all PSSs. On the other hand, optimizing parameters are gain constant (*K*) and lead-lag time constants (*T*_1_, *T*_2_, *T*_3_, and *T*_4_).

The entire systems are modelled based on linear-time-invariant (LTI) state-space (SS) theory around an operating point from a set of nonlinear differential equations. The linearized system model is represented in terms of the state matrix (*A*), input matrix (*B*), output matrix (*C*), and feed-forward matrix (*D*) shown in

(1) $$\begin{array}{c} \dot{x}=A\cdot x+B\cdot u,\\ y=C\cdot x+D\cdot u, \end{array}$$

where *x* is the vector of state variables and *u* is the vector of input variables. The eigenvalues determined from state matrix *A* reflect the overall system stability. Therefore, objective functions are formulated to move eigenvalue forcefully from unstable region (right side of s-plane) to stable region (left side of s-plane). The key properties of an eigenvalue (*λ* = *σ* ± *jω*) are damping factor (*σ*) and the damping ratio (*ζ*) that are determined from

(2) $$\begin{array}{c} \sigma_{i}=\text{real}\left(\lambda_{i}\right),\\ \zeta_{i}=-\frac{\sigma_{i}}{\sqrt{\sigma_{i}^{2}+\omega_{i}^{2}}}, \end{array}$$

where *i* = 1, 2, 3,…, *n* and *n* is the total number of eigenvalues in the power system. The objective functions are maximized or minimized in order to achieve system stability under constraints of damping controller parameters shown

(3) $$\begin{array}{c} K_{\min}\leq K\leq K_{\max},\\ T_{1,\min}\leq T_{1}\leq T_{1,\max},\\ T_{2,\min}\leq T_{2}\leq T_{2,\max},\\ T_{3,\min}\leq T_{3}\leq T_{3,\max},\\ T_{4,\min}\leq T_{4}\leq T_{4,\max}. \end{array}$$

## 3. Objective Functions

**Figure 3:**
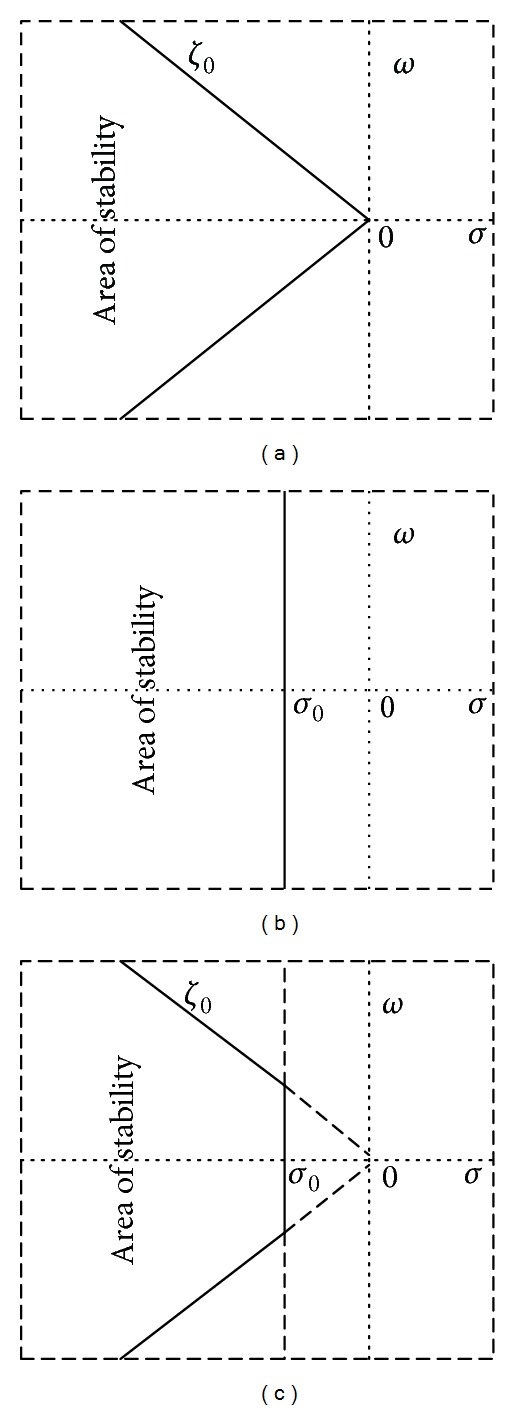
Stability regions defined by different objective functions in s-plane.

The main purpose of objective function formulation is to shift eigenvalues from unstable region to stable region shown in Figure 3 to achieve sufficient damping over power system oscillations. The popular objective functions are described in (4)–(8).

### 3.1. Single Objective Functions

These types of objective functions are formulated by focusing one property of eigenvalues (either *σ* or *ζ*) that partially ensure system stability for total *j* operating points. From research paper [5–8], the objective function is constructed to improve only damping factor shown in (4). In this case, the objective function *J*_1_ is set to minimize

(4) $$\begin{array}{c} J_{1}=\max\left\{\text{real}\left(\lambda_{ij}\right)\right\}. \end{array}$$

Damping ratio is a measure of damping oscillations. In [9], the eigenvalue with poorest damping ratio is selected to maximize *J*_2_ shown in

(5) $$\begin{array}{c} J_{2}=\min\left(\zeta_{ij}\right). \end{array}$$

In other literature [10], CDI is considered as the objective function that considered all dominant modes to achieve maximum damping ratios instead of one mode only. CDI is described by (6). Here, *J*_3_ is minimized in order to optimize controller parameters.

(6) $$\begin{array}{c} J_{3}=\overset{n}{\underset{i=1}{\sum }}\left(1-\zeta_{ij}\right). \end{array}$$

### 3.2. Multiobjective Functions

Different approaches are adopted to move eigenvalues into D-shaped stability regions. Multiobjective functions in (7) [11, 14–18] and in (8) [12, 19] are reported to minimize (*J*_4_) and maximize (*J*_5_), respectively, in order to achieve D-shaped stability criteria:

(7) $$\begin{array}{c} J_{4}=\overset{np}{\underset{j=1}{\sum }}\sum_{\sigma_{ij}\geq \sigma_{0}}{\left(\sigma_{0}-\sigma_{ij}\right)}^{2}+a\cdot \overset{np}{\underset{j=1}{\sum }}\sum_{\zeta_{ij}\leq \zeta_{0}}{\left(\zeta_{0}-\zeta_{ij}\right)}^{2}, \end{array}$$

(8) $$\begin{array}{c} J_{5}=-\max\left(\sigma_{ij}\right)+\min\left(\zeta_{ij}\right). \end{array}$$

## 4. Differential Search Algorithm

Differential search algorithm (DSA), as a metaheuristic optimization algorithm, is selected to assess the objective functions (4)–(8) for improving damping performances by multimachine PSSs. Tuning the parameters of PSSs damping controllers is a multimodal optimization problem and DSA is recommended specially to solve this type of problem [20]. In DSA, the initial population of organisms is generated using (9) and their movements are characterized by (10):

(9) $$\begin{array}{c} x_{nm}=\text{lo}{\text{w}}_{m}+\left(\text{u}{\text{p}}_{m}-\text{lo}{\text{w}}_{m}\right)\cdot \text{rand}, \end{array}$$

(10) $$\begin{array}{c} x_{\text{new},nm}=x_{nm}+R\cdot \left(x_{d}-x_{nm}\right), \end{array}$$

where *x*_*nm*_ is the initial population and low_*m*_ and up_*m*_ are the lower and upper constraint vectors of PSS parameters, respectively. The scale factor *R* is generated using a gamma random function as shown in

(11) $$\begin{array}{c} R=\text{rand}g\cdot \left[2\cdot \text{rand}\right]\cdot \left(\text{rand}-\text{rand}\right), \end{array}$$

where rand*g* and rand are the gamma random function and uniformly distributed pseudorandom number, respectively. In DSA, donors are considered to be located in fruitful area. Therefore, generated populations are moved towards the location of donors and donors are made up by reshuffling the original populations as shown in

(12) $$\begin{array}{c} x_{d}={\left(x_{nm}\right)}_{\text{random}\_\text{reshuffling}}. \end{array}$$

**Figure 4:**
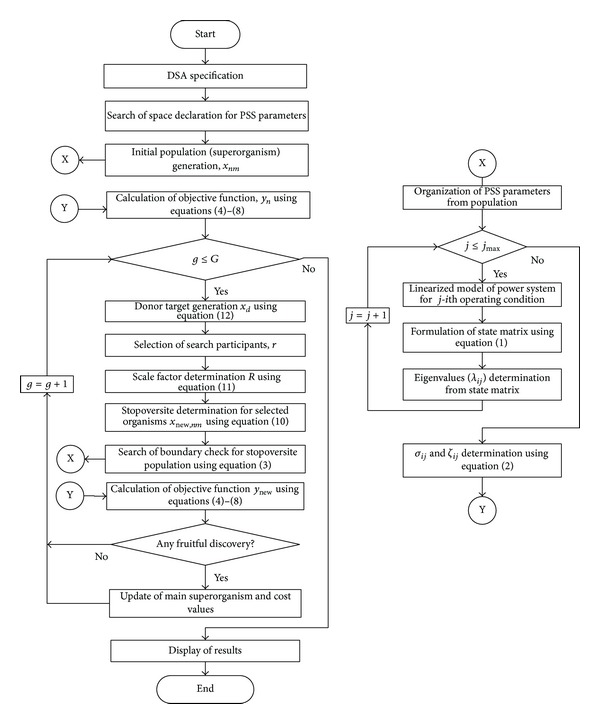
Flow chart of PSS parameters optimization with different objective functions using DSA.

For a fair comparison, the simulation settings of DSA are kept the same for each objective function. The simulation settings are mentioned in the appendix. To understand the overall procedures of this research, the step by step works are depicted in flow chart diagram shown in Figure 4. Interested authors are referred to the original paper of this optimization algorithm in [20].

## 5. Results and Discussion

In order to evaluate the effectiveness of selected objective functions in (4)–(8), simulations are performed in linearized model and nonlinear model of the power system. The linear model simulations are performed using DSA technique to minimize or maximize the objective functions that concurrently bring unstable and poorly damped electromechanical modes into stable region. The common base setting for optimization simulations using DSA is the same (mentioned in the appendix) for all objective functions. All the simulations are performed on a desktop computer with 3.14 GHz processor and 4 GB DDR3 RAM. The system operating points are considered as mentioned in Table 1. The test system has four PSSs and total 20 parameters are to be optimized.

Simulations for each objective function have been performed 10 times and data are recorded. Then the best one (according to the objective function value) is selected for comparison. The same process is executed for all five objective functions. The best optimized parameters of four PSSs are listed in Table 2. From the best optimized value, the major dominant modes are determined as shown in Table 3 with corresponding damping ratios and frequencies. Sensitive modes are highlighted (bold) for easy understanding.

For objective function *J*_1_, although dominant modes have achieved expected damping ratios, (0.15) it may lead to system instability. The reason is its interarea mode's damping factor, which is below expected value (−1.5). In addition, the frequency of interarea modes is very low; therefore it is enough to show poor damping. For remaining objective functions, it is noticeable that dominant modes for only *J*_4_ are achieved expecting damping factor and damping ratio. Moreover, *J*_4_ has no modes with very low frequency. The improvement is based on the damping factors and damping ratios. Single objective functions *J*_1_, *J*_2_, and *J*_3_ do not have both expected values, which is a sign of poor damping performance. Whereas multiobjective functions *J*_4_, *J*_5_ have better performance compared with single objective functions. Even though *J*_5_ do not have expected damping factor for its low frequency mode, which may lead to poor damping.

**Figure 5:**
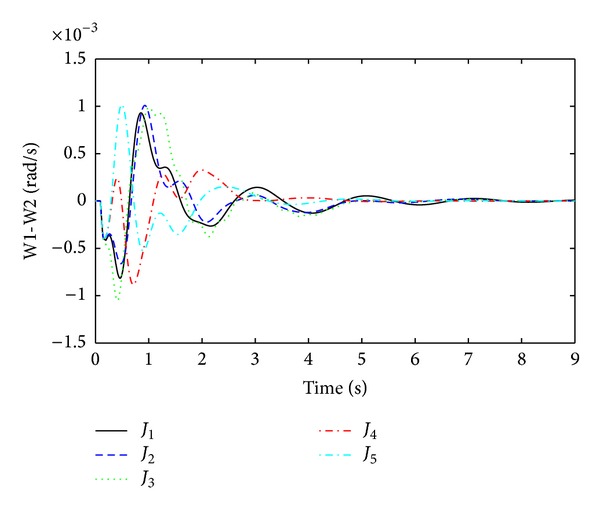
Local mode of oscillations between G1 and G2 (W1-W2) for three-phase fault at bus 9.

**Figure 6:**
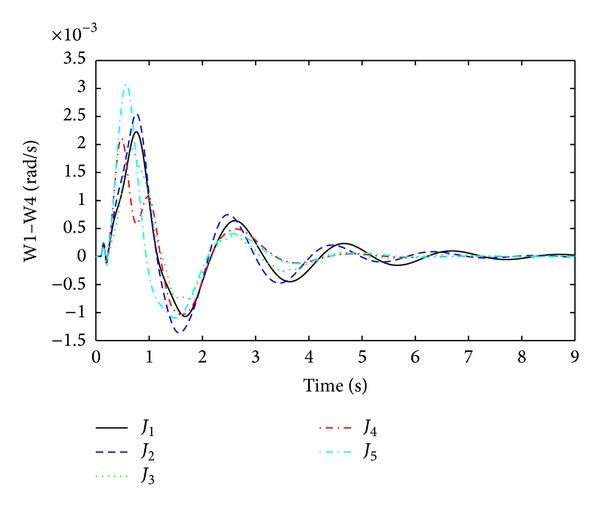
Interarea mode of oscillations between G1 and G4 (W1–W4) for threephase fault at bus 9.

The objective functions performances are assessed further in nonlinear time domain simulations by comparing damping performance. In order to check the robustness of damping controller, it is important to evaluate damping performances under considered (Table 1) and not considered operating points during linear model optimization. As a considered operating condition, three-phase fault near bus 9 (between lines 9 to 13) and, as a not considered operating point, three-phase fault near bus 13 (between lines 13–9) are executed individually. For both conditions, faults are applied for 100 ms duration and cleared by opening remote end of the faulted line at 150 ms. After that the system is operating in only one tie line and oscillations between generators to generators start growing. Optimized PSSs sense the oscillations and provide damping to suppress it. The quality of a good damping controller is measured on how fast the settling time is achieved and how smoothly the overshoot it can handle to avoid major damages by oscillations. Figures 5 and 6 are the responses of local mode and interarea mode oscillations obtained for fault at bus 9. In Figure 5, it is observed that only *J*_4_ has lower overshoot and very fast settling time than others. For interarea mode of oscillations in Figure 6, *J*_4_ and *J*_5_ have almost the same settling time except high overshoot for *J*_5_.

**Figure 7:**
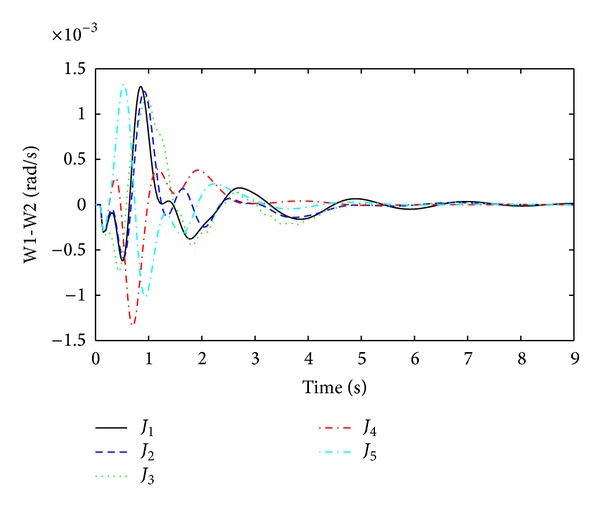
Local mode of oscillations between G1 and G2 (W1-W2) for three-phase fault at bus 13.

**Figure 8:**
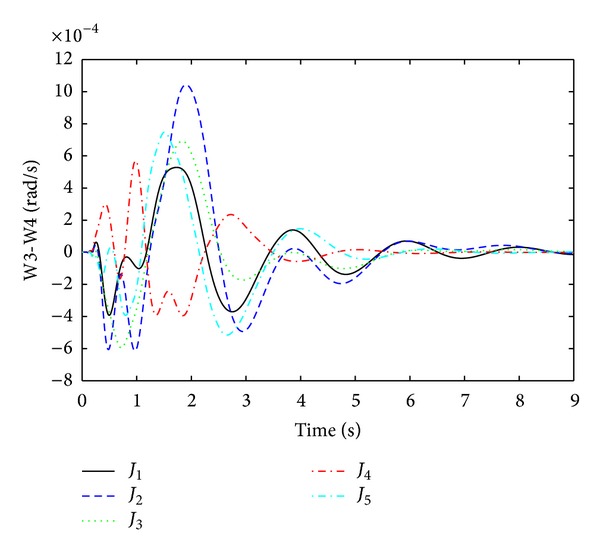
Local mode of oscillations between G3 and G4 (W3-W4) for three-phase fault at bus 13.

**Figure 9:**
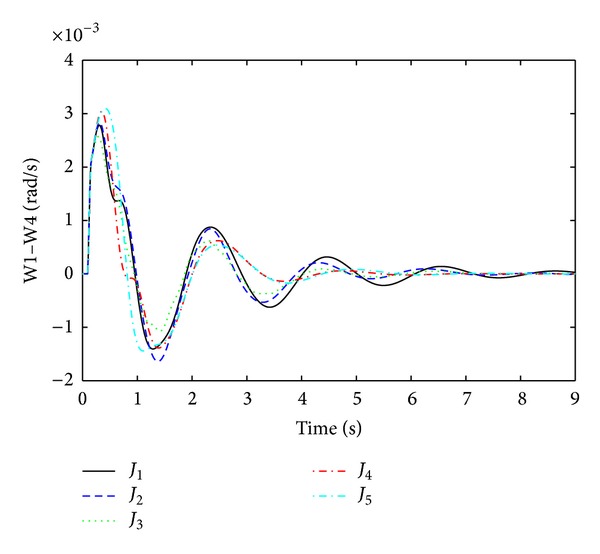
Interarea mode of oscillations between G1 and G4 (W1–W4) for three-phase fault at bus 13.

**Figure 10:**
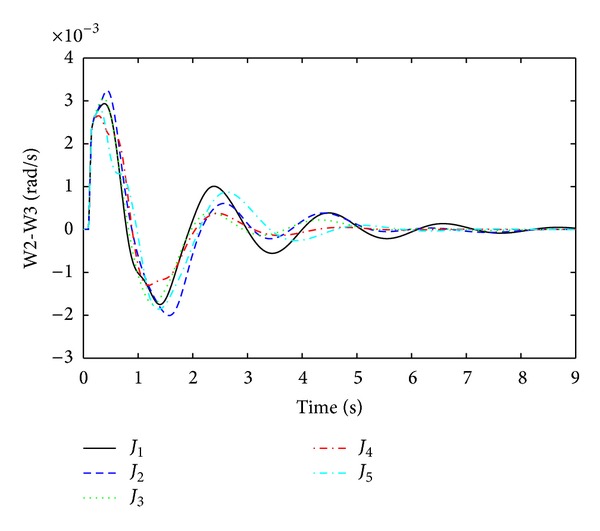
Interarea mode of oscillations between G2 and G3 (W2-W3) for three-phase fault at bus 13.

Rotor oscillation responses for the fault at bus 13 are obtained as shown in Figures 7, 8, 9, and 10. From Figures 7–10, it is observed that single objective functions *J*_1_ and *J*_2_ have very poor settling times and overshoot than other. Objective function CDI has quite a satisfactory damping performance except in Figure 7. For *J*_4_, the best settling times are achieved in all modes and the overshoot values are also quite reasonable. Multiobjective function *J*_5_ has damping performance just like single objective functions *J*_1_, *J*_2_. Thus, from Figures 7–10, the time domain simulations are used to validate the optimization performance in order to check the system stability for the three-phase fault at bus 13.

## 6. Conclusion

In this research, comparative analyses of different objective functions used for power system oscillation damping controller design have been performed. Damping power system oscillations by optimal tuning of PSS is approached. Metaheuristic algorithm DSA is kept the same in settings for all objective functions. Simulations for each objective function are conducted 10 times and best results are selected for comparison. The comparisons are performed in terms of dominant eigenvalues (damping factor, damping ratio) and the damping performance in nonlinear time domain simulations. The comparative results show that the multiobjective D-shaped stability function is a more efficient objective function than that of others. The performance of damping oscillation is further verified in nonlinear time domain simulations. Therefore, problem formulation into D-shaped objective function is recommended in order to design robust damping controller. Further researches should be conducted to develop more efficient objective functions reflecting better performance of damping controllers. Formulation of new and improved objective functions should be challenged based on more extensive comparative analysis with existing uses.

## Figures and Tables

**Table 1:** System operating conditions considered in damping controller design.

Case	Operating points	Faults at bus	Between buses
1	Base case (all line in services)		
2	Three phase fault	9	9 and 13
3	Line to line fault	13	13 and 10

**Table 2:** Best optimized parameters for each objective function using DSA.

Objective functions		*K*	*T*_1_	*T*_2_	*T*_3_	*T*_4_
*J*_1_	PSS1	5.1903	1.1099	0.2358	1.6706	1.3299
	PSS2	7.2073	1.1549	1.2313	0.7452	1.5635
	PSS3	6.3461	0.8985	0.5746	0.8453	0.3337
	PSS4	10.7097	0.0906	0.7335	1.4030	1.6083

*J*_2_	PSS1	5.0422	0.9573	1.8312	1.6562	0.2857
	PSS2	29.3894	0.2130	1.7013	0.0165	1.9849
	PSS3	21.8602	0.2569	0.1395	1.3798	1.1226
	PSS4	25.9223	0.0634	1.7467	0.9554	0.8587

*J*_3_	PSS1	15.0137	1.3111	1.0455	0.4944	0.0597
	PSS2	40.2497	0.2833	1.8740	0.5664	1.9577
	PSS3	31.1375	0.7925	0.9937	0.1303	0.0108
	PSS4	22.8605	0.2659	1.6025	1.0201	1.6150

*J*_4_	PSS1	25.5890	1.3868	0.9446	0.0788	0.7359
	PSS2	3.10292	0.7043	0.7753	1.3975	0.1023
	PSS3	12.5965	0.0191	0.5753	1.1611	0.4992
	PSS4	16.5014	0.6357	0.3429	1.0108	0.9893

*J*_5_	PSS1	33.794	0.0411	1.9849	0.7937	1.1574
	PSS2	31.8907	0.3248	0.0561	0.3886	1.8845
	PSS3	28.9673	1.1705	0.7381	0.1170	0.0134
	PSS4	35.8091	1.1773	0.8512	0.0769	0.6321

**Table 3:** Comparative improvement in dominant modes for different objective functions.

Objective functions	Dominant modes eigenvalues	Damping ratio (ζ)	Frequency (ω)
*J*_1_	−0.7180 − 3.8405*i*	0.1838	**0.6112**
	−2.0831 − 10.7434*i*	0.1904	1.7099
	−3.2354 − 17.3224*i*	0.1836	2.7570

*J*_2_	−0.7376 + 4.0867*i*	0.1776	**0.6504**
	−2.2613 + 12.3786*i*	0.1797	1.9701
	−3.0685 + 17.0267*i*	0.1774	2.7099

*J*_3_	−1.6484 − 4.8225*i*	0.3234	0.7675
	−2.6479 + 18.0621*i*	**0.1451**	2.8747
	−0.7421 + 18.5663*i*	**0.0399**	2.9549

*J*_4_	−2.7588 − 11.0712*i*	0.2418	1.7620
	−4.4346 + 13.9203*i*	0.3035	2.2155
	−2.7934 + 17.2958*i*	0.1594	2.7527

*J*_5_	−1.2487 − 3.3262*i*	0.3515	**0.5294**
	−2.5901 − 6.5999*i*	0.3653	**1.0504**
	−3.8229 − 16.0199*i*	0.2321	2.5496

## Appendix

Simulation settings of DSA: DSA control parameter, P1 = P2 = 0.3 × rand, population size, *n* = 40; problem dimension, *m* = 20, and epoch size = 300.

Objective functions settings: expected damping factor, *σ*_0_ = −1.5, expected damping ratio, *ζ*_0_ = 0.15.

## References

[1] Kundur P (1994). *Power System Stability and Control*.

[2] Rogers G (2000). *Power System Oscillations*.

[3] Kundur P, Klein M, Rogers GJ, Zywno MS (1989). Application of power system stabilizers for enhancement of overall system stability. *IEEE Transactions on Power Systems*.

[4] Hannan MA, Chan KW (2006). Transient anlysis of FACTS and custom power devices using phasor dynamics. *Journal of Applied Sciences*.

[5] Abido MA (2001). Parameter optimization of multimachine power system stabilizers using genetic local search. *International Journal of Electrical Power & Energy Systems*.

[6] Abido MA (1999). Novel approach to conventional power system stabilizer design using tabu search. *International Journal of Electrical Power & Energy Systems*.

[7] Abdel-Magid YL, Abido MA, Mantawy AH (2000). Robust tuning of power system stabilizers in multimachine power systems. *IEEE Transactions on Power Systems*.

[8] Mostafa HE, El-Sharkawy MA, Emary AA, Yassin K (2012). Design and allocation of power system stabilizers using the particle swarm optimization technique for an interconnected power system. *International Journal of Electrical Power & Energy Systems*.

[9] Linda MM, Nair NK (2013). A new-fangled adaptive mutation breeder genetic optimization of global multi-machine power system stabilizer. *International Journal of Electrical Power & Energy Systems*.

[10] Cai L-J, Erlich I (2005). Simultaneous coordinated tuning of PSS and FACTS damping controllers in large power systems. *IEEE Transactions on Power Systems*.

[11] Abdel-Magid YL, Abido MA (2003). Optimal multiobjective design of robust power system stabilizers using genetic algorithms. *IEEE Transactions on Power Systems*.

[12] Alkhatib H, Duveau J (2013). Dynamic genetic algorithms for robust design of multimachine power system stabilizers. *International Journal of Electrical Power & Energy Systems*.

[13] Farsangi MM, Kyanzadeh S, Haidari S, Nezamabadi-pour H (2010). Coordinated control of low-frequency oscillations using real immune algorithm with population management. *Energy Conversion and Management*.

[14] Abd-Elazim SM, Ali ES (2013). A hybrid particle swarm optimization and bacterial foraging for optimal power system stabilizers design. *International Journal of Electrical Power & Energy Systems*.

[15] Shayeghi H, Shayanfar HA, Jalilzadeh S, Safari A (2010). Multi-machine power system stabilizers design using chaotic optimization algorithm. *Energy Conversion and Management*.

[16] Yassami H, Darabi A, Rafiei SMR (2010). Power system stabilizer design using Strength Pareto multi-objective optimization approach. *Electric Power Systems Research*.

[17] Khodabakhshian A, Hemmati R (2013). Multi-machine power system stabilizer design by using cultural algorithms. *International Journal of Electrical Power & Energy Systems*.

[18] Eslami M, Shareef H, Taha MR, Khajehzadeh M (2014). Adaptive particle swarm optimization for simultaneous design of UPFC damping controllers. *International Journal of Electrical Power & Energy Systems*.

[19] Khaleghi M, Farsangi MM, Nezamabadi-Pour H, Lee KY (2011). Pareto-optimal design of damping controllers using modified artificial immune algorithm. *IEEE Transactions on Systems, Man and Cybernetics C: Applications and Reviews*.

[20] Civicioglu P (2012). Transforming geocentric cartesian coordinates to geodetic coordinates by using differential search algorithm. *Computers & Geosciences*.

